# AP92-like Crimean-Congo Hemorrhagic Fever Virus in *Hyalomma aegyptium* Ticks, Algeria

**DOI:** 10.3201/eid2202.151528

**Published:** 2016-02

**Authors:** Matej Kautman, Ghoulem Tiar, Anna Papa, Pavel Široký

**Affiliations:** University of Veterinary and Pharmaceutical Sciences Brno, Brno, Czech Republic (M. Kautman, P. Široký);; University Chadli Bendjedid, El Tarf, Algeria (G. Tiar);; Aristotle University of Thessaloniki, Thessaloniki, Greece (A. Papa);; Central European Institute of Technology, Brno (P. Široký)

**Keywords:** tickborne disease, Algeria, Hyalomma aegyptium, Crimean-Congo hemorrhagic fever virus, CCHFV, viruses, Crimean-Congo hemorrhagic fever, ticks, parasites, zoonoses

**To the Editor:** Crimean-Congo hemorrhagic fever virus (CCHFV) (Nairovirus, *Bunyaviridae*), the causative agent of Crimean-Congo hemorrhagic fever, has been detected in sub-Saharan Africa, southeastern Europe, the Middle East, and central Asia. The virus has been detected in >31 species of ticks and is transmitted to humans by bite of infected ticks (mainly of the genus *Hyalomma*) or by contact with body fluids or tissue of viremic patients or livestock. The disease is characterized by fever, myalgia, headache, vomiting, and sometimes hemorrhage; reported mortality rate is 10%–50% (*1*).

CCHFV strains currently constitute 7 evolutionary lineages, 1 of which (Europe 2) contains the prototype strain AP92, which was isolated in 1975 from *Rhipicephalus bursa* ticks collected from goats in Greece (*2*). This strain seems to have low or no pathogenicity for humans; only a few mild cases have been reported (*3*). This observation is supported by the relatively high (14.4%) seroprevalence but no clinical cases in humans in northwestern Greece (*4*). The documented tick carriers of this strain are *R. bursa* and *Hyalomma marginatum* (*5*).

*Hyalomma aegyptium* ticks are highly host specific; adults feed almost entirely on tortoises of the genus *Testudo* (*6*) and occassionally on hedgehogs and hares. Unlike adult ticks, the larvae and nymphs are less host specific and feed on a wide spectrum of hosts (e.g., other reptiles, birds, and mammals [including humans]) (*7*). This trait elevates the epidemiologic role of the tick as a possible bridge vector connecting wildlife, domestic animals, and humans.

To determine the biological and epidemiological role of *H. aegyptium* ticks, during 2009–2010, we collected 56 adult ticks from 12 *Testudo graeca* tortoises at a locality near the city of Aflou in Laghouat Province, Algeria. We tested the ticks for probable CCHFV infection by using nested reverse transcription PCR (*8*), which amplifies a partial fragment of the CCHFV small RNA segment. We slightly modified the assay: reverse transcription time was 60 minutes and annealing temperature was 52°C (*9*). 

In total, 16 (28.6%) ticks were positive for CCHFV. The PCR products of 15 (26.8%) positive samples were sequenced. BLAST (http://blast.ncbi.nlm.nih.gov//Blast.cgi) analysis identified all 15 sequences as CCHFV with 98%–100% identity to the AP92 strain (GenBank accession no. DQ211638). Two variants of AP92 were detected and differed by 0.6%. A phylogenetic tree was constructed by Bayesian inference, using MrBayes version 3.1.2 (http://mrbayes.sourceforge.net/index/php) under a general time-reversible plus gamma distribution plus invariable site model with 10^7^ generations setup (Figure). Sequences are available in GenBank (accession nos. KT99097 and KT99098).

**Figure:**
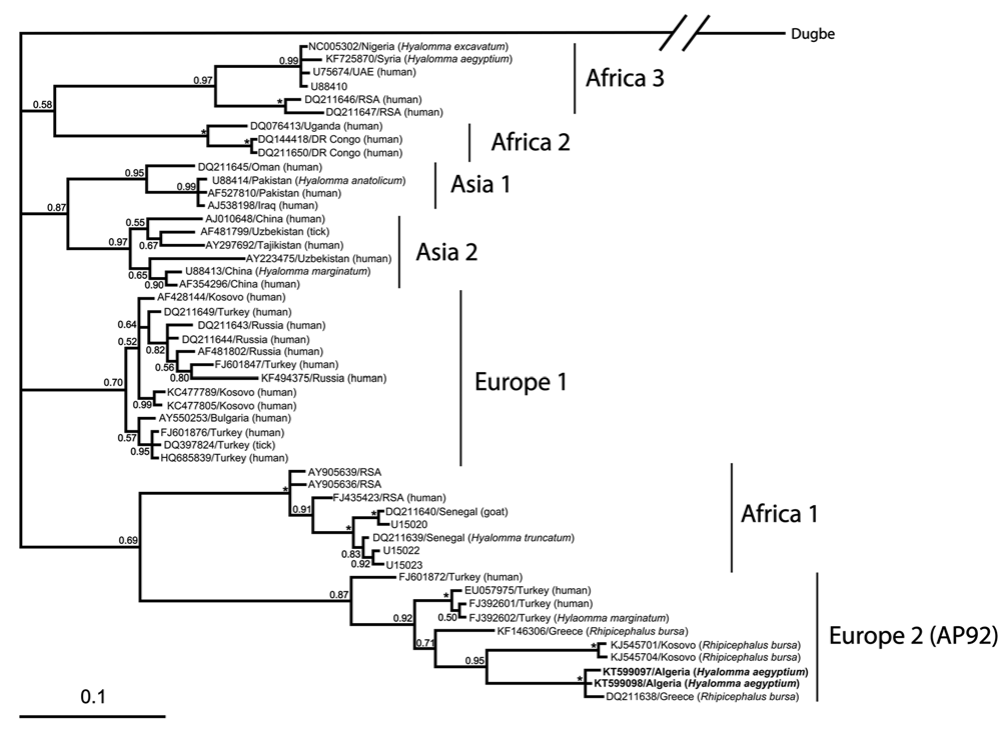
Phylogenetic analysis of Crimean-Congo hemorrhagic fever virus small RNA segment sequences, performed by using Bayesian inference in MrBayes version 3.1.2. (http://mrbayes.csit.fsu.edu/) under a general time-reversible plus gamma distribution plus invariable site model with 10^7^ generations setup. Bootstrap values (>50%) are shown at nodes. Asterick (*) indicates 1.00 bootstrap value. Scale bar represents the estimated number of substitutions per site. Individual sequences are named with GenBank accession number/country of origin and the host, if available, in parentheses. Boldface indicates sequences of virus isolated from ticks collected from 12 *Testudo graeca* tortoises in Algeria, 2009–2010.

Our findings demonstrate the presence of CCHFV in Algeria, either recently introduced or overlooked. The nearest location where CCHFV has been reported is the Zouala region in Morocco, where the virus was detected in *H. marginatum* tick larvae and nymphs collected from migratory birds (*10*). It also confirms association of AP92-like sequences with *H. aegyptium* ticks.

This study shows that the Europe 2 lineage is not restricted to the Balkan region and Turkey. The role of *H. aegyptium* ticks as CCHFV vectors should be further tested. Further investigation of the distribution of CCHFV in ticks in Algeria is also needed. To date, CCHFV strains of lineage Europe 2 have not been associated with severe disease in humans. However, physicians in Algeria should be aware of potential Crimean-Congo hemorrhagic fever cases.
